# Goniosynechialysis combined with cataract extraction for iridoschisis: A case report

**DOI:** 10.1097/MD.0000000000008295

**Published:** 2017-10-20

**Authors:** Zhipeng You, Yan Qin, Guodong Li, Ke Shi

**Affiliations:** Department of Ophthalmology, the Second Affiliated Hospital, Nanchang University, Nanchang, Peoples’ Republic of China.

**Keywords:** cataract extraction, goniosynechialysis, iridoschisis, secondary glaucoma

## Abstract

**Rationale:**

Iridoschisis is a rare eye disease. In this case report, we described the examination and diagnosis of a case of iridoschisis accompanied by secondary glaucoma. We also observed the effects of treating the patient with a combination of goniosynechialysis and cataract removal.

**Patient concern:**

A 67-year-old female patient presented with decreased vision in both eyes. An eye examination indicated that visual acuities (VAs) were 20/100 and light perception in the right and left eyes, respectively. Both eyes exhibited shallow anterior chambers and narrow angles. The lower portion of the iris was loosened, and cable-like tissue was visible. The intraocular pressures in the right and left eyes were 22 mmHg and 58 mmHg, respectively. At the time of presentation, no medication was being used.

**Diagnoses:**

The patient was diagnosed with iridoschisis [oculus sinister (OU), indicates left eye], secondary glaucoma (OU), senile cataract (OU), and pterygium (oculus uterque, indicates both eyes).

**Intervention:**

After relevant examinations were conducted, goniosynechialysis and phacoemulsification with intraocular lens implantation were performed on the right eye under local anesthesia.

**Outcomes:**

Two days after surgery, the right eye had VA of 20/40 and a transparent cornea. The anterior chamber was deeper, and intraocular pressure had decreased to 16 mmHg. Three months after surgery, the patient exhibited improved VA in the right eye and a lower IOP of 11 mmHg.

**Lessons:**

Relative to other approaches, goniosynechialysis combined with cataract removal is a better treatment option for iridoschisis complicated with closed-angle glaucoma triggered by peripheral anterior synechiae.

## 1 Introduction

In 1922, Schmitt first reported iridoschisis, which he referred to as iris splitting.^[1]^ In 1945, Forster suggested that this condition involves the actual splitting of the iris mesoderm in a region into 2 layers and termed it iridoschisis.^[2]^ The etiology of iridoschisis remains unclear. Iridoschisis is generally thought to be caused by atrophy of the iris stromal layer caused by aging or diseases involving vascular occlusion that are accompanied by iris stromal degradation. There is a hidden cavity between the anterior and posterior lobes of the iris stroma; moreover, there are no anterior hyaloid membranes in the iris crypts. Therefore, when the flexibilities of the iris stroma and the vessels decrease, preventing these components from functioning in conjunction with the pupillary sphincter and the dilator muscle, the tissue of the iris stroma gradually splits. In addition, aqueous humor can flow into the cavity between the anterior and posterior lobes of the iris stroma because of an external force that causes the cavity to expand, resulting in iris splitting.^[3]^

Iridoschisis is often found among patients 60 to 70 years of age and typically involves both eyes with no family history. Most commonly, iridoschisis is found underneath the iris and involves separation between the anterior iris stroma and the “posterior iris stroma and muscle.” The loose anterior iris tissues atrophy and degrade into a fibrous form. One side is fixed at the ciliary muscle and extrudes forward, whereas the other side floats in the anterior chamber and can touch the corneal endothelium, causing edema in the corresponding region.^[4]^ Approximately 65% of patients with iridoschisis may present with various complications, including different types of glaucoma, keratoconus, syphilitic interstitial keratitis, microphthalmos, and lens dislocation.^[5]^

## 2 Case report

**Figure 1:**
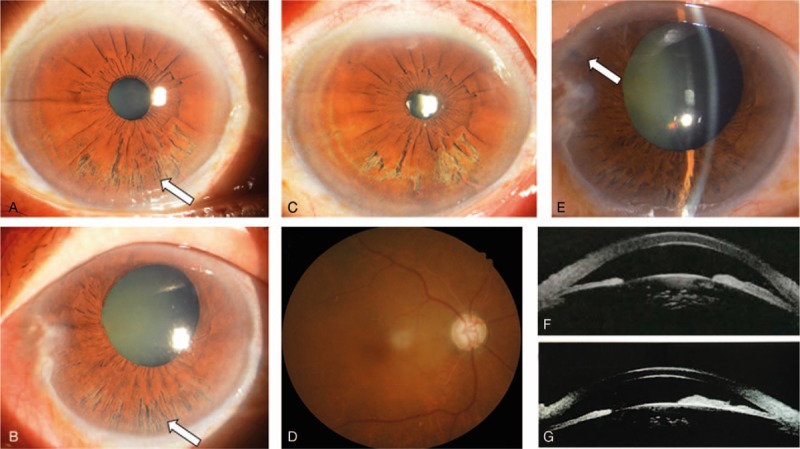
(A) Presurgical photograph of the anterior segments of right eye. (B) Presurgical photograph of the anterior segments of left eye. (C) Postsurgical photograph of the anterior segments of right eye. (D) Fundus photography of right eye taken after surgery. (E) Photo of the anterior segment of the left eye after LPI. The white arrow points to the LPI cut. (F) Presurgical ultrasound biomicroscopy (UBM) image of the left eye. (G) Postsurgical UBM image of the left eye.

A 67-year-old female patient sought treatment at our department because of decreased vision without pain in both eyes for the prior 6 months. Her medical, family, and psychosocial history included no abnormal conditions. An eye examination indicated that visual acuities (VAs) in the right and left eyes were 20/100 and light perception, respectively, and vision in both eyes was noncorrectable. The right eye exhibited ciliary congestion, but the cornea was clear, with 2530 corneal endothelial cells/mm^2^. The central anterior chamber (AC) depth was 3 corneal thicknesses (CTs), and the AC angle was N IV (Scheie classification). Peripheral anterior synechiae were detected in 3 out of 4 quadrants via dynamic gonioscopy. The lower portion of the iris was loosened, and cable-like tissue was visible (Fig. 1A). The pupil had a diameter of approximately 3 mm and was unresponsive to light. Opacities were found in the lens cortex, and ocular fundus images were blurry. The disc color was slightly pale, and the cup/disc (C/D) ratio was approximately 0.8. The left eye exhibited ciliary congestion. Nasal conjunctival hyperplasia had invaded 1 mm past the limbus and was accompanied by mild corneal edema, with 1910 corneal endothelial cells/mm^2^. The central AC depth was 3 CT, and the AC angle was N IV (Scheie classification). Peripheral anterior synechiae were found in all quadrants via dynamic gonioscopy. The lower portion of the iris was loosened, and cable-like tissue was visible (Fig. 1B). The pupil had a diameter of approximately 6 mm and was unresponsive to light. Opacities were found in the lens cortex, and the ocular fundus was fuzzy. The intraocular pressures in the right and left eyes were 22 mmHg and 58 mmHg, respectively.

After differential diagnoses of progressive iris atrophy (the iridocorneal endothelial syndrome subtype), acute primary angle-closure glaucoma, and Axenfeld-Rieger syndrome, the patient was diagnosed with iridoschisis [oculus sinister (OU), indicates left eye], secondary glaucoma (OU), senile cataract (OU), and pterygium [oculus uterque (OS), indicates both eyes]. After relevant examinations were performed, goniosynechialysis and phacoemulsification with intraocular lens implantation were conducted on the right eye under local anesthesia. Pupil dilation took longer than the average pupil dilation time for cataract patients. During phacoemulsification, we found the condition to be similar to intraoperative floppy iris syndrome, and no rupture of the iris was detected during surgery.

Two days after surgery, an eye examination indicated that the right eye had VA of 20/40 and a transparent cornea. The anterior chamber was formed, and the intraocular lens was in place (Fig. 1C). Ocular fundus images were clear (Fig. 1D). An examination of the left eye produced the same results as those obtained before surgery. The intraocular pressures in the right and left eyes were 16 mmHg and 44 mmHg, respectively.

For financial reasons, the patient elected to postpone surgical treatment on the left eye. However, laser peripheral iridotomy (LPI) was conducted, and the patient was provided with 4 intraocular pressure-lowering drugs (pilocarpine, timolol, brinzolamide, and brimonidine). A follow-up examination performed 1 month after surgery indicated that the right eye had VA of 20/33 and a transparent cornea. The anterior chamber was formed, and the intraocular lens was clear. The left eye had VA of light perception and exhibited reduced corneal edema. The AC depth was 3 CT, and the LPI cut was clear (Fig. 1E–G). Opacities were found in the lens cortex, and the ocular fundus was fuzzy. The intraocular pressures in the right and left eyes were 12 mmHg and 28 mmHg, respectively. Continued use of the 4 antiglaucoma eye drops was prescribed.

Three months after surgery, the patient's right eye had an improved VA of 20/33, and VA in the left eye was light perception. Similar signs were observed for both eyes at the most recent follow up. The intraocular pressures in the right and left eyes were 11 mmHg and 23 mmHg, respectively.

The Ethics Committee of the Second Affiliated Hospital of Nanchang University approved the above study and supervised the privacy rights of human subjects.

## 3 Discussion

Iridoschisis is clinically uncommon, with disease onset typically occurring between 60 and 70 years of age. The characteristics of iridoschisis are progressive splitting and separation of the anterior and posterior layers of the iris stroma without the formation of holes. In typical cases, bilateral lesions are found underneath the iris.^[6]^ Careful assessment is required to differentiate iridoschisis from progressive iris atrophy (subtype of iridocorneal endothelial syndrome),^[7]^ acute primary angle-closure glaucoma(APACG),^[8]^ and Axenfeld-Rieger syndrome.^[9]^ The main clinical characteristics of progressive iris atrophy are ectopia pupillae, severe iris stromal atrophy, and holes in all layers of the iris. Progressive iris atrophy also typically manifests in only 1 eye and is often found in young-to-middle-aged adults.^[7]^ The iris stromal atrophy observed in APACG is segmental and is typically found above the iris and near the pupil margin. Axenfeld-Rieger syndrome is a type of congenital glaucoma that is bilateral but generally detected in teenagers. The typical change in the iris is that the papillary sphincter shows clear uplifting of a brown or orange-yellow ring with a dark brown feathery outer edge in the pupillary sphincter region. Structural damage to all layers of the iris and the pupillary sphincter can be observed in certain patients. The patient in this case showed clinical characteristics consistent with iridoschisis.

Approximately half of iridoschisis patients present with either open- or closed-angle glaucoma. Open-angle glaucoma is triggered by pigment released because of iris splitting or obstruction in the flow of aqueous humor caused by blockage of the trabecular meshwork by fragments of the iris stroma. There are 2 mechanisms underlying the onset of closed-angle glaucoma. First, this condition could result from pupillary block. Second, closed-angle glaucoma can be produced by peripheral anterior synechiae and angle closure triggered by intruding split-off iris stromal tissue.^[10]^ The patient in this case was diagnosed with iridoschisis complicated with closed-angle glaucoma. Closed angles were observed on UBM at admission. The morphology of the iris was flat, with no apparent up-lifts. The main reason for angle closure was peripheral anterior synechiae rather than pupillary block. AC depth did not significantly increase after exploratory laser peripheral iridectomy, reaching only 1.70 mm compared with a presurgical depth of 1.59 mm. Intraocular pressure also did not significantly decrease after this procedure, further supporting the speculation detailed above.

Goniosynechialysis combined with cataract removal is commonly utilized to treat acute and chronic PACG.^[11–12]^ This procedure can allow peripheral anterior synechiae to be effectively detached. When combined with cataract removal, goniosynechialysis can permit widening and opening of the anterior chamber angle to increase anterior chamber depth and lower the intraocular angle. The patient in this case had peripheral anterior synechiae and opacities in the lens cortex. Therefore, viscoelastic agents were used to mechanically separate peripheral anterior synechiae. Given the results of an opened anterior chamber angle and the restored flow of aqueous humor, the patient's treatment was satisfactory.

In summary, our patient's medical history and presenting symptoms were consistent with iridoschisis with secondary glaucoma. However, cases involving this condition are rarely observed; therefore, detailed slit-lamp examination is required, and iridoschisis must be differentiated from progressive iris atrophy, APACG and A-R syndrome, among other possibilities. The prognosis observed after treatment suggests that laser peripheral iridectomy may provide limited treatment for iridoschisis complicated with closed-angle glaucoma triggered by peripheral anterior synechiae. Goniosynechialysis combined with cataract removal is a better treatment option. In this case report, only 1 patient was studied, which was a limitation of our study, and we will conduct a multicenter investigation of this therapeutic approach in future work.
